# Smart nanosystems for disease-resistant potatoes: a new Frontier in nanobiotechnology

**DOI:** 10.1039/d5ra07976d

**Published:** 2026-03-17

**Authors:** Bo Chen, Mohammad Umair Rafiq, Muhammad Usman, Sarmad Frogh Arshad, Akhtar Hameed, Manzar Abbas

**Affiliations:** a School of Agriculture, Forestry and Food Engineering, Yibin University Yibin 644000 China; b Institute of Plant Protection, Muhammad Nawaz Shareef University of Agriculture Multan 66000 Pakistan; c Department of Biochemistry and Biotechnology, Muhammad Nawaz Shareef University of Agriculture Multan 66000 Pakistan; d Inner Mongolia Saikexing Institute of Breeding and Reproductive Biotechnology in Domestic Animal Hohhot China abbas2472@hotmail.com; e National Center of Technology Innovation for Dairy Hohhot 010020 China

## Abstract

Microbial diseases in potato crops pose significant threats to production quality and crop protection. Yield losses due to these diseases are unsustainable for a world that is increasingly reliant on potato-based diets. Conventional management strategies, including chemical and biological controls, have been employed, but they often disrupt biodiversity conservation. The resulting ecological degradation has driven researchers to seek sustainable alternatives. Emerging technologies now address phytopathogens—viruses, fungi, and bacteria—while minimizing the adverse effects of traditional methods. Among these, nanotechnology, utilizing materials with at least one dimension between 1–100 nm, has revolutionized plant health through nanopesticides and targeted pesticide delivery systems. These innovations exhibit minimal ecological impact while demonstrating potent antimicrobial activity against key potato diseases such as early blight, late blight, common scab, soft rot, and blackleg. Nanoparticles (NPs) generate reactive oxygen species (ROS), which lyse microbial cells while simultaneously activating defense-related signaling pathways (salicylic acid and jasmonic acid pathways). These pathways upregulate pathogenesis-related (PR) genes, enhancing PR protein synthesis to combat microbial invasion. Nanotechnology has enabled the design of advanced nano-biosensors for disease detection. By leveraging nanoparticle properties such as a high surface-area-to-volume ratio, photoluminescence, electrical conductivity, and biomolecular interaction, these sensors precisely identify microbial biomarkers. Additionally, the small size, surface charge, controlled release, and tunable surface chemistry of nanoparticles help in optimizing targeted gene and drug deliveries. Nanotechnology further enhances genome-editing tools, like CRISPR/Cas9 and RNA interference (RNAi), facilitating the development of disease-resistant transgenic potato varieties. It also induces the production of antioxidant enzymes, osmolytes, stress-responsive genes, and structural barriers to mitigate abiotic stresses. In summary, nanotechnology offers a multidisciplinary approach for combating phytopathogens, ensuring sustainable potato cultivation with minimal ecological disruption.

## Abstract 2

Microbial diseases in potato crops pose significant threats to production quality and crop protection.

## Graphical abstract

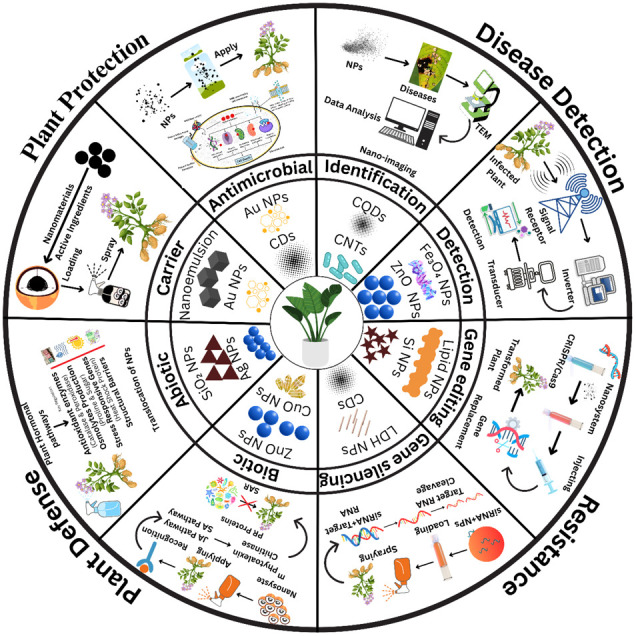

## 1 Introduction

Potato belongs to the Solanaceae family of plants with the scientific name *Solanum tuberosum* L.; it bears tubers in the underground part of the soil and has been recognized as one of the most produced and consumed crops globally after wheat, rice and maize.^1^ Some European countries, like Ireland, have adopted potato as a staple food because of its reasonably high yields and rich nutrient profile. The presence of nutritional components such as potassium, vitamin C, vitamin B6, proteins, thiamine and niacin in potatoes increase their essentiality as these components are important for maintaining human health. The climate and soil resilience of the potato crop ensures its availability across the globe.^2^ Nowadays, the potato crop has valorized the global food industry due to its nutritive and economic value. The food processing industries of potatoes have been transformed extensively into production units of value-added products. By-products like potato starch are used in soups, sauces, gravies and puddings and as binding agents and texture enhancers in meats and baked items. The production of alcoholic beverages and fermentation into spirits due to their high starch content has also supported the alcohol industry. Potatoes have recently emerged as a viable source of bioplastics as they are developed into biodegradable packaging materials that replace traditional plastic packaging, and this is a broader trend towards sustainability and for decreasing the environmental impacts of plastic trash.

### 1.1 Potato productivity under siege

The production of potatoes was estimated to be approximately 375 million tonnes, while its global consumption was approximately 236 million metric tonnes based on the latest statistics of FAO in 2023. China is the largest producer of potatoes, followed by India, Germany, France, the Netherlands, the UK, Belgium, Russia, Ukraine, the USA, Canada, Argentina and Australia. Asia has been a major contributor to the production of staple food crops like wheat, rice and potatoes. Potato is the second most targeted crop in Asia after wheat, rice and maize. In Asia in 2022, potatoes were harvested in an area of approximately 10 336 800 hectares with a production of approximately 203 338 000 tons. China, the world's potato superpower, accounts for more than 20% of the global potato-growing area and harvest. China produces approximately 92 million metric tons of potatoes every year, ∼66 kg per capita. Based on its contribution to world potato production, we can say that if there is a drought in China that affects potato output, this might have consequences for the entire world food supply. India is the next largest potato producing country, with a production of approximately 50 million metric tons every year. Several other Asian countries including Pakistan, Bangladesh, Iran, Japan and Turkey are among the top 20 producers of potatoes. Most of the world would go hungry without potatoes from Asia.

As per the Trade Development Authority of Pakistan (TDAP), Pakistan contributes around 4% to world potato production, while according to the Pakistan Economic Survey 2022-23, the total potato cultivating area was around 341 000 hectares, producing 8 320 000 tons in the country. In 2023-24, potato production was estimated at 8 441 000 tons using the cultivated area of 339 000 hectares, an increase in production by 1.5%. Potato production in Pakistan is mainly focused in Punjab and then in KPK, Balochistan and Sindh. Two major regions of Pakistan renowned for potato cultivation are Punjab and Gilgit Baltistan. In Punjab, potato production was estimated to be over 8.1 million tons, which was harvested from an area of around 808 000 hectares.

Considering the production figures of potatoes, the lion's share of this production is lost at the pre- and post-harvest stages due to diseases or other factors. Potato diseases have remained a major concern for improving potato production in Pakistan. Thus, for better production, protection is necessary. Potatoes are not only consumed directly by consumers, but different value-added products of this crop have increased its importance.^3^ As the world relies on this crop, it is necessary to grow safe and healthy crops with high production yields which are often disturbed by different biological problems such as attacks by pathogens.^4^

### 1.2 The invisible yield thief

Potato has remained below the threshold of different vulnerable pathogens including viruses, bacteria, fungi and nematodes. These pathogens not only affect the production of the crop but also deteriorate the quality of tubers. Considering the importance of this crop, it is necessary to protect it from destructive microbes. Blackleg, ring rot, soft rot, zebra chip, pink eye, bacterial wilt and common scab are important bacterial diseases of potatoes that cause huge losses of the crop. Fungal diseases like early blight, late blight, brown leaf spot, black scurf, silver scurf, stem canker, black dot, skin spot, gangrene, verticillium wilt, wart disease, violet root rot, and watery wound rot have negative impacts on potato quality and quantity. Potato Virus Y, Potato Virus X, Potato leaf roll virus, Potato mop top virus, Tobacco necrotic virus, Tobacco rattle virus, and latent and mild mosaic viruses damage the crop aggressively. Root knot nematodes, potato cyst nematodes, and sting nematodes affect the potato crop vigorously. These diseases not only destroy its quality and yield but also affect its market value indirectly. All major diseases (bacterial, viral, fungal and nematode) of potato are mentioned in Table 1.

**Table 1:** Key phytopathogens of potato: causative agents, symptoms and chemical treatments

Diseases	Pathogens	Symptoms	Treatment	Ref.
Blackleg of potato	*Pectobacterium atrosepticum*	Blackening and rotting of the stem starts from the base upward; leaves become yellow, wilt and show premature senescence	Mercuric chloride, Agristep- or Semesan bel copper-based fungicides like Mancozeb	13–15
Ring rot of potato	*Clavibacter michiganensis*	A soft cheese-like rotting of the vascular ring is the earliest sign, hence the term “ring” rot. In severe cases, it completely rots and cracks the tuber's skin	Roccal, zephiran, hydrogen peroxide (5–20%)	16, 17 and 18
Soft rot of potato	*Dickeya solani*	Soft rot symptoms often include soft and moist tissues of tubers that are cream-colored	5-Nitro-8-hydroxyquinoline, chlorine dioxide	19 and 20
Zebra chip disease	*Candidatus Liberibacter*	Chlorosis and purpling of foliage, upward rolling and burning of leaves, stunting and the development of aerial tubers	Thiamethoxam, fludioxonil, difenoconazole	21, 22 and 23
Pink eye disease	*Pseudomonas fluorescens*	Pink but slightly elevated regions on damp. The afflicted areas are typically located around the eyes and at the tuber stem end. Often present on freshly dug tubers but harder to detect on dried ones	Chlorothalonil, copper-based fungicides	24, 25 an 26
Bacterial wilt of potato	*Ralstonia solanacearum*	Wilting and yellowing of leaves and stunted plants. Vascular browning is ubiquitous, with bacterial ooze splashing from the incision	Methyl bromide, 1,3-dichloropropene, metam sodium with chloropicrin	27, 28 and 29
Common scab of potato	*Streptomyces scabiei*	Slight protrusions with sunken centers may occur and be covered with tiny amounts of corky tissue. Scab layers are transformed into lesions	Kasugamycin, copper-based fungicides	30, 31 and 32
Brown rot of potato	*Ralstonia solanacearum*	Symptoms emerge with brown staining of vascular rings that leads to rotting. Plant wilts with immediate effects	Copper nitrate, sodium hydroxide, magnesium sulphate polyvinylpyrrolidone	33, 34 and 35
Aerial stem rot of potato	*Pectobacterium* spp.	Black to brown slimy decay on potato stems	Copper hydroxide famoxadone	36, 37 and 38
Early blight	*Alternaria solani*	Lesions appear on leaves with a targeted spot having concentric rings. These frequently occur few weeks after emergence as extremely black or brown patches on lower leaves that eventually coalesce	Azoxystrobin, difenoconazole	39, 40 and 41
Late blight	*Phytophthora infestans*	Tuber skin becomes darker brown, while the internal rot becomes reddish brown. Granular rot may remain at the surface or spread to the center of the tuber	Zorvec endavia, ranman, revus, mancozeb	42, 43 and 44
Brown leaf spot	*Alternaria alternata*	On leaves, it produces little dark brown necrotic tissue patches with dark brown edges. Starting as tiny lesions, the spots might merge to cover a significant portion of the leaf or petiole surface	Fossil (azoxystrobin + difenoconazole), topsin (thiophanate methyl)	45, 46 and 47
Black scurf	*Rhizoctonia solani*	Affected tubers may exhibit growth abnormalities that resemble glyphosate damage and exhibit slowed growth in conjunction with stronger brown skin patches known as netting or elephant hide	Pencycuron, flutolanil, iprodione	48, 49 and 50
Silver scurf	*Helminthosporium solani*	Small silvery gray specks expand into circles with a slightly darker boundary. These circles expand and join together. Unwashed tubers with silver scurfs often have a sooty look due to spore formation	Pentachloronitro-benzene	51, 52 and 53
Stem canker	*Rhizoctonia solani*	Plants remain stunted and have folding of upper leaves. Brown, slightly recessed lesions with definite edges develop at the stem base and stolons. If the lesions are severe, they may unite and girdle the stem	Tolclofos-methyl + thiram, fosetyl-aluminium	54, 55 and 56
Black dot	*Colletotrichum coccodes*	Vascular disease may trigger stem wrapping, leading to foliar wilt. The affected skin looks light brown to undamaged but with spots. Later, boring dark brown spots may form	Fluazinam, azoxystrobin	57, 58 and 59
Skin spot	*Polyscytalum pustulans*	Dark grey and slightly elevated spots on tubers. A mature fungal lesion typically creates an empty ring around a rising center. Lesions may appear singly or in bunches, and they can be randomly scattered or localized around the eyes, stolons, and injured skin		60, 61 and 62
Gangrene	*Phoma exigua* var. *Foveata*	The potato has discolored, dark sunken and irregularly shaped patches. Gangrene also produces interior decay and cavities filled with sparse gray or yellow mould	Fluazinam	63, 64 and 65
Verticillium wilt	*Verticillium dahliae*	The disease is frequently recognized as scattered patches in a field and can cause stunting, premature plant senescence and diminished output. Plants lose turgor and wilt on hot sunny days	Chlorothalonil, propiconazole, thiophanate-methyl, azoxystrobin	66, 67 and 68
Wart disease	*Synchytrium endobioticum*	Galls (wart-like growths) start out green, change brown and eventually black. Potato wart is seen at the time of harvest. Tubers have abnormal wart-like growth, ranging in size from a pin to a fist	Famoxadone + cymoxanil, tolclofos methyl + thiram, dimethomorph + copperoxychloride, mandipropamid + mancozeb, zoxamide + mancozeb	69, 70 and 71
Violet root rot	*Rhizoctonia crocorum*	Infected roots and tubers develop distinctive red-violet mycelium, which is occasionally accompanied by little black sclerotia. The mycelium then penetrates the tube	Flutolanil, fludioxonil	72 and 73
Watery wound rot	*Pythium ultimum*	Affected flesh may be grey to brown in color with a dark border. It is damp and soon liquefies	Metalaxyl, mancozeb	74, 75 and 76
Potato stem end rot	*Fusarium oxysporum*	A slightly sunken circular lesion appears with corky rot on the potato surface at the end of the stem	Mefenoxam	77, 78 and 79
Potato virus Y	Aphid	Symptoms include leaf deformation, mottling and crinkling, chlorotic mosaics in haulm, vein necrosis, reduced leaflet size and overall reduction in vigour	Thiamethoxam, (25 WG @ 150 g ha^−1^	80, 81 and 82
Potato virus X	Mechanical transmission	Mosaic, chlorosis, necrotic lesions and decreased leaf size are morphological symptoms	Ribavirin	83, 84 and 85
Potato leafroll virus	Green Peach Aphid	In the upper leaves, a slight rolling and red/orange tinge may occur. The bottom leaves may roll and become dry and brittle, having a papery feel. The upper leaves upturn and show yellowing. Plants remain stunted	Pyrethroids, carbamate, organophosphates, neonicotinoids	86, 87 and 88
Potato mop top virus	*Spongospora subterranea*	Dark arcs and rings on the tuber skin are visible. The lower leaves have yellow spots, and the upper leaves are pale green. Stunted plants may bunch upper leaves with wavy/rolled margins (hence mop top). Tubers may be distorted, russetted, and cracked and may have net-like surface patches	Fluazinam, cyazofamid	89, 90 and 91
Tobacco necrotic virus	*Olpidium brassicae*	Dark brown and increased patches on leaves later to dark sunken lesions in ring/horseshoe form	Cross protection	92, 93 and 94
Tobacco rattle virus	*Trichodorus* spp.	The infection starts with brown flecking and arcs that are concentric. Stems become stunted with mottled leaves	Abamectin, azoxystrobin	95, 96 and 97
Mosaic virus	Aphids	Poor growth, yellowing, stunting, mottling or mosaic patterns and plant deformities are the symptoms	Thiamethoxam (25 WG @ 150 g ha^−1^	98, 99 and 100
Root knot nematode	*Meloidogyne incognita*	Form swelling on roots known as galls, which are small in size, while egg masses appear on feeder roots. Wilting appears on above ground parts	Cadusafos	101, 102 and 103
Potato cyst nematode	*Globodera rostochiensis*	Form cysts on roots, while above-ground symptoms include plant stunting, flower delaying, leaf yellowing and ultimate wilting	Fluazaindolizine	104, 105 and 106
Sting nematode	*Belonolaimus* spp.	They are linked with severely misshapen, abnormally russetted and scruffy tubers. Wilting and stunting of plants are the ultimate effects	Fluensulfone, 1,3-dichloropropene	106, 107 and 108

**Fig. 1:**
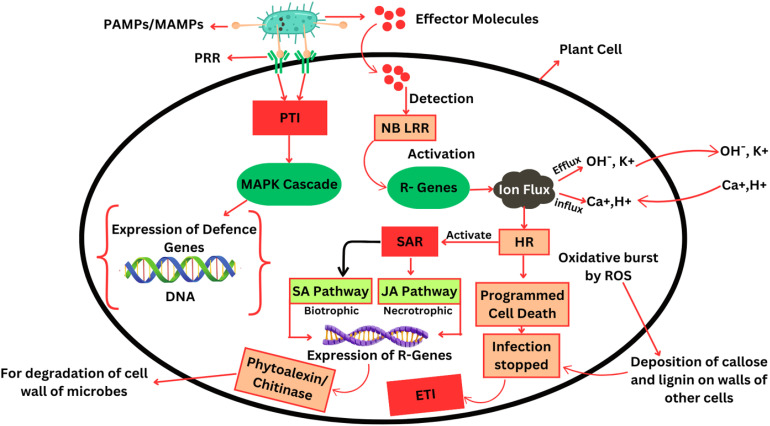
Molecular mechanisms of plant immune response activation against pathogens. Pathogen-associated molecular patterns (PAMPs/MAMPs) are recognized by plant pattern recognition receptors (PRRs), initiating a defense cascade. This includes MAPK (mitogen-activated protein kinase) signaling, which upregulates the expression of defense-related genes (*e.g.*, phytoalexins and chitinases) to degrade microbial cell walls. Upon pathogen effector detection, a hypersensitive response (HR) is activated, often leading to localized programmed cell death to restrict biotrophic and necrotrophic pathogens. The salicylic acid (SA) pathway predominantly defends against biotrophic pathogens, while the jasmonic acid (JA) pathway combats necrotrophic invaders, ensuring a dynamic and tailored immune response.

Different management strategies, like cultural, physical, biological and chemical, have been applied against such microbial load over time, but these strategies do not eliminate the risk of pathogens. Chemical control measures have detrimental effects on both plant and consumer health. On the contrary, the continuous application of chemical pesticides leads to the development of microbial resistance. Such detrimental effects of pesticides have led researchers to develop resistant potato cultivars with enhanced yield traits using genetic engineering techniques to mitigate the effects of pathogens (Fig. 1).

### 1.3 Recognition gap: undetected pathogen effector and its toll on plant defense

Micro-organisms present on or within plants produce various signals based on volatile chemical compounds, hormones, hormone mimics, proteins and carbohydrates.^5^ Microbial life depends on signals derived from proteins and carbohydrates that are often described as Microbe or Pathogen-Associated Molecular Patterns (MAMPs or PAMPs).^6^ Plants produce distinct Pattern Recognition Receptors (PRRs) localized in the plasma membrane that bind MAMPs/PAMPs and regulate plant immunological responses. PAMP/Pattern Triggered Immunity (PTI), also known as basal resistance, is the first line of defense in which most plant species limit pathogen infection in response to MAMPs/PAMPs.^7^ In the meantime, microbes release effector molecules during invasion, which are essential components of pathogenesis. Plants produce a series of immune reactions known as Effector Triggered Immunity (ETI) that recognize effector molecules.^8^ Plants that do not identify these effector molecules become susceptible and cannot activate their defense mechanisms.^9,10^ In the case of the detection of effector molecules, plants activate their defense systems, like the expression of *R* genes, production of antimicrobial proteins like phytoalexins, closure of stomatal pathways, over-production of reactive oxygen species (ROS) and expression of hypersensitive response (HR), followed by programmed cell death.^11^ With the expression of HR, plants may undergo systemic acquired resistance (SAR) through signaling pathways (jasmonic acid (JA) and salicylic acid (SA) pathways). Through these signals, plants activate *R*-genes, which encode pathogenesis-related (PR) proteins, to combat pathogens.^12,14^

### 1.4 Rising threat: agrochemical-resistant pathogens in global potato security

Microbial resistance refers to the ability of bacteria, fungi and viruses to endure agents, like antibiotics or pesticides, that normally kill them. Microbial resistance develops through various mechanisms initiated by pathogens.^109^ Pathogens continue to develop infections leading to severe diseases like late blight or black leg, which reduce crop yields. Therefore, high doses of chemicals or additional applications are applied to control resistant strains that increase production costs and environmental damage. For sustainable potato production and food security, the fight against microbial resistance is necessary.^110^ The resistance of potato pathogens against synthetic pesticides is caused by the presence of different mechanisms by which pathogens survive and thrive in the presence of chemical treatments. To be able to develop appropriate management strategies, it is important to understand these mechanisms in depth.^111^ Here are some key mechanisms of microbial resistance:

#### 1.4.1 Genetic mutations

Genetic mutations in pathogens alter their response to chemicals, which may lead to harm to the target sites of pesticides, making these treatments useless. For example, a fungus can possess an enzyme that the fungicide ought to target, but the morphology of this enzyme is altered by genetic mutation, so the fungus can grow and multiply unharmed.^112^

#### 1.4.2 Efflux pumps

Efflux pumps are proteins that actively translocate toxic substances from the cell across the membrane. The efflux pump may extrude out the chemicals or make them less potent through biofilm formation and quorum sensing molecules. This mechanism enables pathogenic viability in the presence of lethal concentrations of treatment agents.^113^

#### 1.4.3 Biofilm formation

Biofilms are protective layers of polysaccharides produced by associated microorganisms around bacterial and fungal communities, protecting them against biotic and abiotic stress. Biofilms act as a barrier against antimicrobials, allowing pathogens to survive and persist on plant surfaces including potato tubers.^114^

#### 1.4.4 Enzymatic degradation

Some pathogens produce enzymes to break down the active ingredients of fungicides and pesticides. For example, some fungi secrete enzymes, like laccase and peroxidase, that break down some chemical compounds, getting rid of their effects and allowing for the pathogen to spread.^115^

#### 1.4.5 Horizontal gene transfer

Horizontal gene transfer allows microbes to secure genes conferring resistance from other microbes. Bacteria can either take on DNA from the environment by transformation, move it to each other by transduction (*via* viruses), or transfer it between two bacteria by conjugation. The genetic exchange may quickly spread resistant traits throughout microbial populations to develop resistance against different pesticides.^116^ Together, these mechanisms contribute to a challenge in managing microbial potato disease and require the development of integrated strategies that decrease reliance on chemical treatment instead of supporting sustainable agricultural practices.

## 2 Nanobiotechnology: a next-generation shield against crop pathogens

The pursuit of sustainable development is vital for safeguarding the availability of essential resources and meeting the basic needs of both present and future populations.^9^ However, microbial resistance to chemical control in food crops has posed serious concerns regarding food safety. The anticipation of biological problems related to food safety has become paramount to feeding the increasing population of the world. A variety of modern cultural, physical, biological and chemical approaches have been developed to ensure the protection of these food crops against microbial resistance. These technologies have revolutionized the traditional world by diverting it into a global village. Nanobiotechnology, the rising technology, consists of two major domains: nanotechnology and biotechnology.^117^ Nanobiotechnology has transformed into a paradigm-shifting technology that has the potential to completely change the 21st century by building a foundation for sustainable and high-quality civilizations.^118^ Nanotechnology may be defined as the use of material particles with at least one dimension of 1-100 nm.^119^ This concept was first introduced in 1959 by an American physicist, Richard Feynman, a Nobel Prize winner, during a meeting of the American Physical Society.^120^ A Japanese scientist, Norio Taniguch, in 1974 proposed the term “nanotechnology” with its proper definition and application, which was given as “nanotechnology is primarily concerned with the separation, consolidation and deformation of materials by a single atom or molecules”.^121^ The other domain, “biotechnology”, refers to the technology used to implement the combination of natural and engineering sciences for the welfare of organisms.^122^ Moreover, nanobiotechnology is referred to as designing and organizing materials on the nanoscale to develop smart devices with extraordinary physicochemical and biological properties and their applications in various fields of life such as agriculture and medicine.^123^

The term nanomaterials (NMs) is an umbrella term that describes materials with a minimum dimension of the nanoscale (1–100 nm), making it a term used to describe nanoparticles, nanocomposites and functionalized nanostructures. In particular, nanoparticles (NPs) refer to discrete nanoscale particles (*e.g.* Ag, CuO, or ZnO particles) that can possess inherent physicochemical or antimicrobial behavior but do not contain engineered complexity. Conversely, nanosystems are engineered nano-architectures where nanoparticles are engineered with other functionalities, like encapsulation, surface engineering, controlled release or targeting. In this review, the term smart nanosystems is used solely to refer to novel nanosystems with advanced or responsive or programmable behavior such as stimulus release (*e.g.*, pH, enzymes, and ROS), stimulus-responsive activation, or delivery to a particular plant tissue or cellular compartment. Based on these, simple metallic nanoparticles lacking these functional characteristics are not considered smart systems but conventional antimicrobial nanoparticles.^124^

### 2.1 Role of nanobiotechnology in agriculture

Nowadays, the commercial use of nanotechniques has increased by merging nanotechnology and biotechnology to make them more functional in industries.^125^ Along with being introduced on an industrial level, nano-biotechnology has had a major impact in agriculture, related to the production and protection of crops. In this way, nanobiotechnology has solved problems such as food availability, for an increasing population.^126^ Products like nano-pesticides, nano-biosensors and nano-fertilizers have been developed to enhance crop production and reduce crop loss caused by microbial attack.^127^ These innovative products ensure sustainable agriculture because they replace synthetic agrochemicals that deteriorate biodiversity. The repetitive use of synthetic fertilizers disturbs the soil structure, texture, fertility and soil microbiota,^128^ while the extensive use of pesticides develops microbial resistance against them. Microbes with developed resistance are more harmful and even lead to epidemic outbreaks. Nano-biosensors are designed to mitigate the vandalism of pesticides by detecting their toxicities.^129^ Microbial resistance is mitigated by delivering anti-microbial agents with the use of nanocarriers. These nanocarriers deliver anti-microbials by releasing them at specific target sites.^130^ Thus, nanobiotechnology greatly enhances crop yield in terms of quality and quantity.

#### 2.1.1 Nanotechnology in plant disease management

The controlled use of nanomaterials makes them strong candidates for replacing pesticide applications. To manage plant diseases, nanoparticles are divided into two categories: either they are directly used as anti-microbials or carriers of pesticides called nanopesticides.^131^ The active ingredients of pesticides are loaded *via* encapsulation inside a nanoparticle shell and released at the target site.^132^ Nanopesticides possess physiochemical properties like stability, solubility, bioavailability, durability, mobility, target specificity, less toxicity and controlled release that make them more efficient and accurate than conventional ones.^133,134^ Efficiency and accuracy in disease management may help to achieve sustainable goals in agriculture. Nanoparticle-based potato disease management can be categorized into three functional types: (i) nanopesticide carriers, (ii) antimicrobial nanoparticles and (iii) immune-activating nanoparticles. These categories have different but complementary contributions to sustainable protection. Nanopesticide vectors are mainly applied to improve the stability, bio-availability and target specificity of pesticides (conventional or biological). Silicon dioxide (SiO_2_) nanoparticles, chitosan nanoparticles, lignin nanoparticles and metal–organic frameworks (MOFs) are the most common nanocarriers. SiO_2_/mesoporous silica nanoparticles (MSNs) are characterized by a high surface area and pore size that can be adjusted to achieve the controlled release of fungicides and bactericides. Chitosan nanocarriers are biodegradable and biocompatible and attach strongly to plant surfaces, which is appropriate for treating leaves and roots. Lignin nanoparticles have a non-toxic, renewable carrier of hydrophobic agrochemicals, and MOFs possess a high loading ability and rapid release. Nonetheless, they cannot be practiced practically due to the cost and manipulation by the regulation.^135^

The interaction of antimicrobial nanoparticles with phytopathogens occurs directly and suppresses them without loading the active ingredients. Some commonly used antimicrobial nanoparticles include silver (Ag), copper (Cu/CuO), zinc oxide (ZnO), magnesium oxide (MgO), and titanium dioxide (TiO_2_). They cause various effects: membrane disruption, ion release, enzyme inhibition and ROS generation, giving them a broad spectrum of action and limiting the resistance chances, but phytotoxicity and environmental persistence must be optimized by dose. The biological behavior of plants exposed to metallic nanoparticles is strongly dependent on the concentration, and it depends on the size of the particles, surface chemistry, plant species, and the stage of development. Although low or optimized doses of Ag-, Cu-, and Zn-based nanoparticles can lead to pathogen suppression and host defense reactions, phytotoxic processes, including oxidative stress disequilibrium, chlorophyll deterioration, root development, membrane harm, and photosynthetic activity, have been linked to excessive concentrations.^136^ Notably, the effective concentration region of pathogens can overlap with concentrations that have adverse biological impacts on plants, meaning that a specific set of metallic nanoparticles has a tight safety margin. Thus, the establishment of crop-specific dose–response relationships and the determination of biologically safe concentration ranges are important to reduce the risk of phytotoxicity and to ensure the viability of the use of nano-enabled disease management tools. The main mechanism of immune-activating nanoparticles is associated with priming or stimulation of the innate defense system of the plant instead of attacking pathogens.^137^ Silica nanoparticles, carbon-based nanomaterials (carbon nanotubes, graphene derivatives), salicylic acid-loaded nanoparticles, and biopolymer nanosystems are the most common immune-activating nanoparticles capable of inducing salicylic acid (SA) and jasmonic acid (JA) signaling, PR gene expression, and systemic acquired resistance (SAR). This immune modulation provides a non-toxic alternative to disease management that is highly chemical-intensive, results in the enhancement of host resistance, and decreases the pressure of a particular pathogen with time. These types of nanoparticles offer a multi-layered disease management solution: pathogen suppression, effective delivery, and immune activation. Future studies must aim at refining the combinations of these nanosystems to be applicable in the fields of long-term safety and regulation acceptance.^138^

**Fig. 2:**
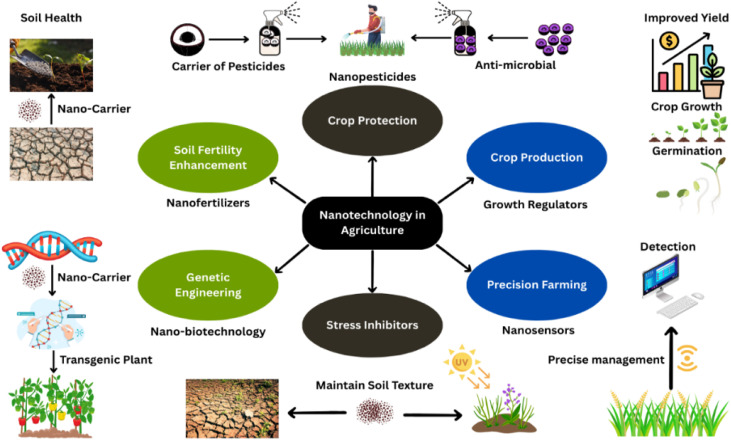
Nanotechnology-driven soil health management for sustainable agriculture. Carrier-mediated pesticide delivery utilizes engineered nanoparticles (NPs) for targeted anti-microbial action, reducing off-target effects and environmental persistence. Soil fertility enhancement integrates nano-enabled growth regulators and precision crop protection strategies to optimize production. Nano-fertilizers leverage smart carriers and genetic engineering to synchronize nutrient release with plant demand. Graphene-based nanobiotechnology enables stress mitigation and precision farming through real-time monitoring. Transgenic plant systems synergize with nanosensors to maintain soil texture and enable data-driven management.

The various nanoparticle systems for potato disease management, as summarized in Table 2, are discussed below to showcase their relative antimicrobial performance and functional application (Fig. 2).

**Table 2:** Case studies of some nanosystems in potato disease management. Reported concentrations are provided as shown in the original studies; the units were not adjusted since the experimental systems did not have equal application methods

Nanosystems	Carrier molecule	Physical properties	Size (nm)	Conc	Diseases	Ref.
CuO/MgO NPs	None/Stabilized *via* green synthesis	High surface area, porous, mesoporous structure	6	3 mg mL^−1^	Potato Brown rot	139
Chitosan NPs	Chitosan polymer	Biodegradable, mucoadhesive, film-forming, hydrophilic	93.76	200 µg mL^−1^	Bacterial wilt	140
ZnO NPs	Stabilized with plant extracts/starch	Photocatalytic, UV-absorbing, piezoelectric, semiconducting	11.5	30 mg L^−1^	Late blight	141
TiO_2_ NPs	Surfactants or polymer matrices	Photocatalytic, UV-active, high chemical stability	20–200	50 µg mL^−1^	Soft rot	142
Ag NPs	Stabilized with plant extract or PEG	High conductivity, plasmonic, antimicrobial	12.7	25 ppm	Early blight	143
MgO NPs	Green synthesis or surfactant-coated	Alkaline, thermally stable, low solubility	52.5–57.3	200 mg L^−1^	Black scurf	144
Fe NMs	Oleic acid or dextran	Magnetic, redox-active, biocompatible	5–100	300 µg mL^−1^	Blackleg	145
rGO-CuO NPs	Reduced graphene oxide (rGO)	High surface area, conductive, hybrid (CuO + graphene)	5–50	1 mg L^−1^	Root rot	146
Cur NPs	Curcumin encapsulated in a polymer matrix	Hydrophobic, antioxidant, fluorescent	45	10 mg mL^−1^	Potato virus Y^NTN^	147
Salicylic acid NPs	Biopolymer (*e.g.*, chitosan) or lipid vesicles	Bioactive, hormone-like, systemic inducer	20–26.6	2.5 mM	Potato leaf roll virus	148
Ag NMs	PEG or polyvinylpyrrolidone (PVP)	Antiviral, surface-active, crystalline	12.6 ± 5	200 ppm	*Tomato spotted wilt virus*	149
Ag NMs	Biopolymer (starch/chitosan)	Stable, antimicrobial, optical-active	55	100 µg mL^−1^	*Root Knot Nematode*	150

The main limitation in the comparison of the antimicrobial efficacy of the nanoparticle-based interventions summarized in Table 2 is that these studies do not always report standardized concentrations. The doses of nanoparticles are quantified in various measures such as mg L^−1^, ppm, µg mL^−1^, and mM, which vary by experimental design, the route of application (foliar spray, soil amendment or hydroponic exposure), and the biological scale. Although aqueous systems can be normalized to a limited degree (*e.g.*, mg L^−1^ = ppm), dosing measures specific to plants cannot be compared quantitatively. Thus, the performance of various nanosystems is viewed qualitatively, focusing on relative performance trends, dose–response behaviour, and the mechanistic mode of action as opposed to absolute concentration values.

Metallic and metal oxide nanoparticles, like Ag, CuO, ZnO and MgO, can be highly effective as antimicrobials with a broad spectrum of activity, mainly *via* membrane disruption, release of ions and generation of reactive oxygen species (ROS). These include silver nanoparticles that are highly effective at relatively low doses against bacterial and fungal pathogens such as early blight and bacterial wilt, but the issue of phytotoxicity requires optimization of the dose. The nanoparticles contain zinc oxide and magnesium oxide, which offer a compromise between antimicrobial activities and environmental friendliness and hence can be used repeatedly in agriculture. The polymeric nanoparticles, especially the chitosan-based systems, have moderate antimicrobial activity but have better performance as delivery systems because they are biodegradable and adhesive to plants and have the capacity to stimulate host defense. Chitosan nanoparticles have particular applications in controlling bacterial wilt and viral diseases when combined with bioactive compounds or genetic materials.^151^ Carbon-based and hybrid nanosystems, including reduced graphene oxide-metal composites, have greater effectiveness due to synergistic effects, which involve physical damage to membranes and the induction of oxidative stress. Altogether, metal-based nanoparticles could be useful for attaining rapid pathogen exclusion, but polymeric and hybrid nanosystems could have better safety, release regulation, and immune-priming properties. These relative distinctions highlight that nanoparticles need to be disease-specific and chosen based on efficacy, levels of phytotoxicity and environmental sustainability as opposed to antimicrobial capabilities alone.^152^

### 2.2 Plant protection: enhancing biosecurity through smart nanosystems

#### 2.2.1 Smart nanobullets: targeting pathogens

In addition to being used as carriers of pesticides, they perform a proper antimicrobial mechanism to suppress the pathogenic effects of pathogens.^153^ Due to their small size, they easily penetrate microbial cells and disturb cellular functions. The overproduction of reactive oxygen species (ROS) leads to oxidative stress, which bursts microbial cells and finally leads to the death of microbes. Some nanomaterials like Ag-NMs and Cu-NMs are known for their antimicrobial behaviour against some potato diseases, like bacterial wilt, soft rot and late blight.^130^ Nanoparticles enter microbial cells and inhibit them from metabolizing in a number of ways, making it difficult for pathogens to become resistant to them.^154^ Particles ranging from 1 to 100 nanometers can adhere to microbial surfaces because of electrostatic properties on their surfaces, thus enabling them to penetrate the cells and penetrate the membrane and cell walls of microorganisms.^155^ In microbial cell membrane interactions, nanoparticles disrupt the selective permeability of cell membranes. For example, silver or zinc (metallic) nanoparticles penetrate and disrupt the lipid bilayer by enhancing its permeability. Cell death and subsequent content leakage may occur due to this disturbance.^156^ A few nanoparticles penetrate microbial cells through the process of endocytosis. Metallic nanoparticles (copper or silver) produce reactive oxygen species (ROS) after they penetrate the cell membrane.^157^ Reactive oxygen species damage the DNA, lipid and protein assembly, leading to oxidative stress that ultimately causes cell death.^158^ stated that DNA is the main target of ROS, which breaks the strands and cross link the protein, base and sugar of DNA. This assessment reveals that the controlled production of ROS is one of the most efficient weapons through which nanoparticles destroy microorganisms.^159^ Some studies have shown that nanoparticles can capture and inactivate essential microbial enzymes. For example, they interact with proteins and enzymes containing sulphur, bringing chemical changes in the metabolic sequences necessary for the development of new microorganisms.^160^ The pathogen is directly weakened and does not create any resistance due to the blockage that affects cellular metabolism. Some kinds of nanoparticles have the unique capability to denature proteins in microbial cells. The pathogen's ability to perform its ordinary metabolic functions is further threatened by this effect on structural and functional proteins for cell survival.^161^

**Fig. 3:**
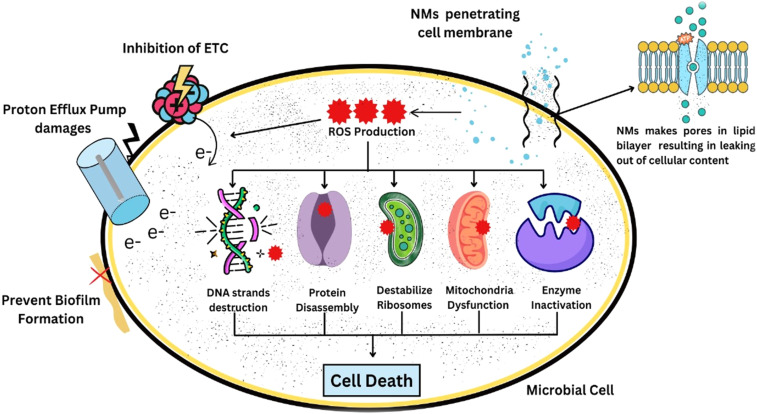
Nanomaterial-mediated microbial inhibition *via* multi-target disruption mechanisms. Electron transport chain (ETC) inhibition through proton efflux pump impairment, preventing biofilm maturation by disrupting redox homeostasis (e^−^ flux blockade). Nanomaterial (NM) penetration induces membrae destabilization, facilitating intracellular reactive oxygen species (ROS) generation (^++^: radical burst; ×: oxidative damage). Genomic and proteomic sabotages occur *via* DNA strand scission, ribosomal destabilization, mitochondrial dysfunction, and targeted enzyme inactivation. Cascade to cell death occur through irreversible biomolecular damage, eradicating microbial viability.

Silver nanoparticles exhibit strong antimicrobial behaviour because they can be used to control bacterial and fungal pathogens, causing common potato diseases such as *Ralstonia solanacearum* (bacterial wilt) or *Phytophthora infestans* (late blight). However, these nanoparticles can destroy microbial cell walls and prevent resistant strains from developing.^140^ For example, ZnO nanoparticles have anti-fungal and antibacterial properties and can be used to control potato diseases. These nanoparticles are also being explored as a way to influence plant immunity and warfare against microbial resistance (Fig. 3).^162^

#### 2.2.2 Surface-tailored nanocoatings for advanced drug transport

Nanomaterials deliver pesticides by carrying them inside the plant at the target site with ease. The active ingredients of pesticides are loaded into the core of nanoparticles, which are easily absorbed by plants due to their small size.^163^ After penetrating the plant cell wall, nanomaterials are released in a controlled form at the site the presence of pathogen. The surface area of active ingredients increases *via* this delivery, which increases their efficiency and stability against diseases of potato.^164^

#### 2.2.3 Surface-engineered nanoparticles in precision drug delivery

**Fig. 4:**
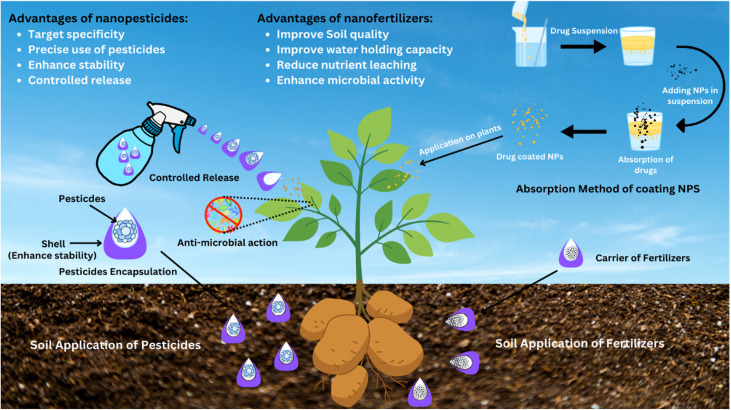
Schematic of nanoenabled agrochemical delivery systems and their multifunctional roles in sustainable agriculture. Nanopesticides are encapsulated within nanoscale shells that enhance chemical stability, enable site-specific targeting, and allow for controlled, sustained release. This nano-engineered approach minimizes off-target dispersion, prolongs antimicrobial activity at both foliar and rhizospheric levels, and reduces environmental dissipation. Similarly, nanofertilizers are depicted as intelligent carriers of essential nutrients, facilitating gradual release and improved bioavailability. These nanostructures enhance soil physicochemical properties, boost microbial dynamics, increase water retention, and significantly limit nutrient leaching, resulting in a transformative strategy for sustainable agriculture that enhances crop yield while minimizing the environmental footprint.

Pesticides control the harmful pest outbreaks of potatoes, but their hazardous concerns are more harmful than their benefits. The widespread and extensive application of pesticides to control phytopathogens not only exerts stress on plants and the environment but also disturbs the economic values of farmers.^165^ Early but precise detection of pathogens is linked with plant protection to implement management strategies. As discussed earlier, nanomaterials are used as carriers for drug delivery or have the potential to inhibit phytopathogens directly.^166^ In drug delivery, the active ingredients of pathogens inhibiting drugs are loaded into the core shell of nanomaterials. That core shell protects drugs and releases them at a specific target gradually. This type of delivery system ensures the controlled and accurate use of drugs at a specific site and time.^167,168^ This method, especially in biocide delivery, facilitates the encapsulation and direct delivery of chemical or biological agents to infected plant tissues or groups of pathogens, thus reducing the dosage needed and preventing resistance development by minimizing the chances of resistance development. NMs increase the stability of the active ingredients of pesticides, as some are degraded and decomposed due to unfavorable environmental conditions.^169^ Antibacterial essential oils, like thymol and carvacrol, are nano-encapsulated to improve their stability, solubility, and bioavailability in potato infection control.^170^ The excessive use of drugs against phytopathogens that put stress on plants is limited, leading to sustainable and precise agriculture (Fig. 4).

By acting as carriers, they increase the life of biopesticides because some are destroyed when exposed to UV light such as avermectin.^171^ Avermectin is loaded into hollow mesoporous silica nanoparticles, which provide a protective shell and release it at a specific site.^172^ Coating NMs with a drug is another method of drug delivery through NPs. In the absorption method of coating NMs, a drug suspension is prepared, and NMs are mixed in the suspension. They absorb the drugs, and when these drug-coated NMs are applied on infected plants, the drugs reach the target sites with ease. Both the drug and NMs activate antagonistic mechanisms against pathogens. The drug coating completely depends on its solubility in liquid dispersion agents, and solubility depends on the presence of an end functional group either in the drugs or the dispersion agent. In addition, NMs deliver fertilizers and nutrients to plants easily.^173^

## 3 Plant disease detection with advanced nanosystems

### 3.1 Nano-biosensors for disease diagnosis

The early detection of plant pathogens effectively helps monitor crop health and develop an integrated disease management strategy. The real-time detection of microbes has become possible, significantly enhancing disease detection due to the intervention of nano-biosensors.^174^ Nano-biosensors are synthesized using nanotechnological methods and are composed of nanomaterials such as nano-probes, carbon quantum dots, carbon nanotubes, nanowires and magnetic nanoparticles. Additionally, inorganic nanoparticles, such as Ag, Au and metal oxide nanomaterials, are generally utilized in their fabrication. Previous studies^175,176^ detected the genomic DNA of *Ralstonia solanacearum*, the causative agent of bacterial wilt in potato, by functionalizing gold nanoparticles (AuNPs) with single-stranded oligonucleotides. These nanobiosensors facilitate the real-time monitoring of plant health and monitor soil environmental conditions, like pH, temperature, humidity and nutrients. They also detect and monitor the pesticide residues, fertilizer concentrations and other agrochemicals in the crop production system.^177^ The advancement of nanobiosensors integrates nano-science, computer science, electronics and biology to make them highly sensitive diagnostic tools. Nanobiosensors function by converting chemical signals into digital data that can be measured and monitored by applying electronic devices.^178^ The fundamental components of nanobiosensors include bio-receptors, transducers and detectors. In the context of plant disease detection, bio-receptors identify microbial signs and symptoms and relay chemical signals to a transducer that converts them into measurable electrical signals. These detectors analyze and amplify these signals into digital data that allow for the precise diagnosis of pathogens.^179^

Historically, the widespread applications of pesticides were employed not only for the management of plant diseases but also inadvertently imposed stress on plant health. However, advancements in plant pathogen detection techniques have enabled significantly precise disease management strategies. Recently, site and target-specific pesticide applications have minimized the adverse effects on plant physiology and overall ecosystem health. The judicious and controlled use of pesticides contributes to biodiversity conservation by minimizing non-target impacts.^180^ Agriculture has significantly proven to be the most affected sector due to the extensive application of agrochemicals, including pesticides, fungicides and insecticides, which often contain heavy metal ions.^181^ These toxic metal ions are introduced to crops and soil, subsequently leaching into water bodies. Upon the consumption of food crops, heavy metal ions accumulate in the human body, posing serious threats due to their carcinogenic nature.^182^ Advanced nanotechnological approaches such as nanobiosensors, have been employed to detect carcinogenic metal ions. In recent advancements, smart nano-biosensors have replaced conventional nano-biosensors for plant pathogen detection and chemical diagnostics. These wearable sensors designed for direct implantation on plant structures such as stems and leaves, exhibit superior flexibility, biocompatibility, cost-effectiveness and lightweight properties, thereby garnering significant interest among phytopathological researchers.^183^

## 4 First line of defense: nano-sensors for early disease detection

Early disease detection avoids the spread of pathogens targeting potato crops.^184^ Biosensors with nanotechnology capabilities identify the presence of a pathogen at extremely low concentrations before the disease spreads, which may require early intervention to prevent loss, thereby reducing the chances of resistance development.^185^ Optical and electrochemical sensors identify some plant stress responses or microbial markers that could be evolved to treat the disease more precisely without the requirement of broad-spectrum antibiotics and postpone drug resistance onset.^186^

### 4.1 Nanoimaging

Nanoimaging has emerged as a revolutionary research technique in plant pathology that enables the identification and examination of plant diseases with nanometer-scale precision. Nanoimaging combines nanotechnology with sophisticated imaging modalities to visualize pathogens, plant tissue responses, and disease development at the cellular and subcellular levels. The ultra-high resolution offered by nanoimaging technologies such as Transmission Electron Microscopy (TEM), Scanning Electron Microscopy (SEM), and Quantum Dot (QD)-mediated fluorescent imaging, enables the early and highly specific detection of plant pathogens weeks before the appearance of overt symptoms.^187^ Underlying nanoimaging is the use of engineered nanomaterials, including metallic nanoparticles (*e.g.*, gold and silver) and semiconductor quantum dots, as biosensing tags or contrast agents. Nanoparticles are typically bound to antibodies, nucleic acids, or ligands with high binding affinity to pathogen-specific markers. Upon injection into infected plant tissues, these nanoprobes bind specifically to targeted structures and generate high-contrast signals in electron- or fluorescence-based imaging systems, thereby enabling direct visualization of pathogenic invasions at ultra-high resolutions.^188^

One of the greatest advantages of nanoimaging for plant disease diagnosis is its ability to detect pathogens during incubation before symptoms appear. This is significant in precision agriculture because early treatment can prevent yield loss and limit the spread of pathogens. For instance, quantum dot-labeled antibodies can efficiently track specific viral or bacterial pathogens in plant tissue at nanometer resolution, which is more sensitive and specific than conventional serological or molecular assays.^189^ Furthermore, nanoimaging methods are crucial for describing structural and functional changes in plant tissue under pathogenic stress. TEM and SEM are among the methods used to provide comprehensive evaluations of cell wall integrity modifications, intracellular transport mechanisms, and organelle morphology in response to pathogen invasion. TEM provides high spatial resolution although it is limited in intact plant samples. Sample preparation includes fixation, dehydration, sectioning, and staining, which can disrupt the original cellular structure and nanoparticle distribution within cells. TEM also provides a two-dimensional ultra-structure from ultra-thin sections, which is not suitable for 3D localization and long-distance transport of nanoparticles in complex plant tissues. Hence, additional imaging techniques such as confocal laser scanning microscopy or X-ray techniques, are required to better understand nanoparticle distribution in plants. Such specificity is crucial for understanding host–pathogen interactions and the generation of disease-resistant crop varieties.^190^

Apart from diagnostics, nanoimaging is also crucial in establishing the administration and efficacy of nanotherapeutics. Following the administration of nanoparticles for the targeted delivery of agrochemicals or immunomodulators into plant cells, tracking can be performed using nanoimaging techniques. Tracking thus far enables precise localization, release kinetics, and interaction with pathogens, ultimately enhancing the efficacy of treatment and minimizing environmental residues.^191^ The integration of nanoimaging methods with computerized analytical systems has enormously amplified their diagnostic capabilities. High-resolution images acquired using transmission electron microscopy (TEM) or fluorescence microscopy can be processed by applying computational algorithms and machine learning schemes, enabling the automation of pathogen detection, measurement of infection intensity, and prediction of disease progression. This fusion of nanotechnology, imaging methods, and artificial intelligence is ready to result in revolutionary changes in plant health monitoring systems.^192^

Although it has many advantages, nanoimaging in plant pathology is not without limitations. Its high cost of equipment, requirement of advanced laboratory facilities, and requirement of highly skilled personnel may limit its application in common farming routines, especially in the developing world. Moreover, the toxicity and environmental hazards of nanoparticles necessitate rigorous risk assessments and the development of biodegradable and biocompatible nanomaterials. To overcome these limitations, recent advances have emphasized the field applicability and miniaturization of nanoimaging platforms. Continuous work on portable imaging systems, lab-on-chip systems, and smartphone-based diagnostic platforms is aimed at enabling the on-site, real-time diagnosis of plant diseases. These technological innovations seek to democratize the accessibility of nanodiagnostics as accessible tools in both cutting-edge research environments and field-level agricultural operations. In short, nanoimaging is an effective and precise tool for plant disease detection, observation, and interpretation.^193^ By providing early, sensitive, and specific imaging of pathogen activities and host responses, nanoimaging significantly enhances the field of plant diagnostics and has great potential for sustainable and intelligent agricultural practices.

## 5 Nanobiotechnology in disease resistance

### 5.1 Genetic approaches in nanobiotechnology

Genetic techniques through nanobiotechnology are being revolutionized to modify plant resistance, especially against microbial diseases. Among the most exciting uses is the use of NPs to create gene delivery systems. Encapsulating these genes allows these NPs to introduce the genes that give plants resistance to different diseases into plant cells more effectively. Nanoparticles can, in turn, be engineered to speed up transformation rates by shielding genetic material from degradation and allowing for absorption in plant tissue. This technique can directly insert beneficial genes into potato plants to make them 10 times more resistant to bacterial, fungal and viral infections. Recently, another significant development in genetic engineering has been the application of CRISPR-nano systems. This method utilizes CRISPR/Cas9 gene editing technology to exactly alter the DNA in the potato plant, which is delivered by nanoparticles.^194^ Scientists can deliver CRISPR components of particular genes to the target cells and modify them to alter their susceptibility to microbial infection. The targeted change is accomplished through the induction of advantageous alleles, or through the knockout of deleterious regulatory genes, to improve plant immunity. The combination of CRISPR technology and nanoparticles simultaneously increases gene editing efficiency while decreasing off-target effects, making the procedure safer and more accurate. However, given all these, there seems to be a lot of opportunity for utilizing nanobiotechnology-based genetic strategies to develop potato cultivars that are more resistant to microbial challenges. CRISPR gene editing combined with nanoparticle-based gene delivery offers a promising strategy for developing resilient crops, thereby reducing the need for chemical treatments and supporting sustainable agriculture. Nanoparticles can deliver RNAi molecules to improve a plant's defense mechanism or silence genes that promote pathogen virulence. This technique reduces the development of microbial resistance by focusing on the infection's use of specific molecular pathways.

### 5.2 Genetic warfare: nanoparticles disrupting microbial resistance

#### 5.2.1 Gene editing

Nanosystems encapsulate and deliver gene-editing materials into plant cells. Lipid nanoparticles (LNPs) facilitate the cytoplasmic delivery of CRISPR/Cas9 through membrane fusion and endosomal escape. Carbon nanotubes (CNTs) enter well and deliver editing agents into protoplasts. Mesoporous silica nanoparticles (MSNs) can deliver plasmid DNA or proteins and can be functionalized for tissue targeting. Polymeric nanocarriers such as chitosan, polyethyleneimine (PEI), and PEG-based polymers stabilize nucleases and gRNA complexes, shielding them from degradation.^195^ These nanocarriers are engineered to penetrate the hard plant cell wall without the use of *Agrobacterium* or biolistics, which tend to insert foreign DNA. CRISPR/Cas9, ZFNs, TALENs, or SHREDDER elements target loci associated with resistance (*e.g.*, StDMR6 and StNLRs). Gene editing agents are encapsulated into nanoparticles through ionic gelatin, self-assembly, or solvent diffusion. Nanoparticles are introduced *via* foliar sprays, vacuum infiltration, or protoplast transfection, circumventing biological barriers to delivering complexes directly into the nuclei or cytoplasm. Targeted cleavage initiates a repair mechanism (NHEJ or HDR), leading to gene disruption (loss-of-function) or targeted sequence insertion (gain-of-function). Edited cells develop into whole plants through tissue culture and are screened for successful edits through PCR, qRT-PCR, and sequencing. This process facilitates transgene-free genome editing, which is more tolerable under regulatory settings and public opinion than transgenic alterations. CRISPR/Cas9 delivered in lipid nanoparticles knocked out the StDMR6-1 gene, enhancing late blight resistance without a yield penalty.^196^ TALENs with silica nanoparticles facilitated accurate edits in virus-resistance genes. SHREDDER systems confirm the edits of polygenic resistance traits by editing multiple copies of a gene.^197^ Gene editing technology and nanosystems are revolutionizing precision agriculture. In potatoes, they enable the quick development of disease-resistant varieties through DNA-free, tissue-specific genome editing. Using advanced delivery methods and changing regulations, the approach holds incredible scope for sustainable crop improvement (Table 3).

**Table 3:** Nanosystems and their roles in nanobiotechnology

Nanosystems	Genome editing tools	Target pathogens	Target gene	Mode of action	Application method	Focus area	Ref.
Carbon quantum dots	CRISPR-Cas9	*Ralstonia solanacearum*	StSR4 (resistance gene)	Disruption of a susceptible gene	Nanoparticle-mediated foliar spray	Bacterial wilt resistance	198
Chitosan nanoparticles	CRISPR-Cas9	Potato virus Y (PVY)	Viral coat protein gene	Virus genome silencing	In planta DNA-free delivery	Virus resistance	199
Gold nanoparticles	CRISPR, RNAi	Fungal/bacterial pathogens	miRNA targets	RNA silencing + gene knockout	Infiltration into potato leaves	Dual protection strategy	200
Silica-based nano-DNA complexes	CRISPR/Cas9	*Pseudomonas syringae*	*S* gene (susceptibility)	Targeted gene disruption	Nanocarrier foliar coating	Bacterial speck resistance	201
Lipid-based nanocarriers	CRISPR-Cas	Soil-borne microbes	*R* genes and effector-binding genes	Priming of immunity	Soil-root zone nanoinjection	Root disease prevention	202
Layered double hydroxide (LDH) nanosheets	Talen, CRISPR	Mixed bacterial load	PPO, StNPR1	Transcription regulation	PEG-mediated protoplast transfection	Stress-resilient potatoes	203
Functionalized nanoparticles	ZFN, CRISPR	General pathogens	GBSSI (starch modulation gene)	Knockout for structural resistance	Somatic cell transformation	Resistance + yield traits	204
Chitosan-CaNPs	CRISPR-Cas9	Potato virus Y	PVY coat protein	RNA interference	Direct leaf infiltration	Broad-spectrum antiviral defense	205
Polymeric nanoparticles	CRISPR	*Phytophthora infestans* (late blight)	Rpi-vnt1	Resistance gene introgression	Agrobacterium + nanoparticles	Blight suppression	206
Chitosan nanoparticles (CS-NPs)	CRISPR-Cas9	*Phytophthora infestans*	StRLK, Rpi-blb2, StWRKY8	Knockout of susceptibility genes; enhanced PR protein expression	Foliar spray/root absorption	Disease resistance engineering	207
Lipid-based nanoparticles (LNPs)	CRISPR-Cas12a	*Pseudomonas syringae*	StDMR6-1	Gene silencing *via* HDR-mediated knock-in	Vacuum infiltration	Bacterial resistance	208
Silica mesoporous nanoparticles (MSNs)	TALEN	*Ralstonia solanacearum* (bacterial wilt)	StSWEET10 (sugar transporter)	Disrupt pathogen nutrient uptake	Soil drench/hydroponics	Metabolic engineering	209
Silica nanocarriers	SHREDER	*Leptinotarsa decemlineata* (beetle)	Male-specific gene disruption	X-shredder gene drive reduces the beetle population, affecting the disease vector	Microinjection into eggs	Pest-based disease vector control	210
Polymeric nanoassemblies	CRISPR/dMac3-Cas9	Fungal pathogens (mixed)	Starch metabolism genes	Multiplex editing to alter tuber chemistry, favoring resistance	Tuber-specific editing	Dual focus: Quality & microbial defense	211
Mesoporous silica nanoparticles	ZFN	Mixed microbial pathogens	StNPR1, StR3a	Activates defense-related transcriptional cascades	Soil root zone inoculation	General resistance boost	212
Nano-clay-based carriers	TALEN + CRISPR mix	*Fusarium* spp.	Defense enzymes (PAL, PR-1)	Enhances pathogen recognition and anti-fungal response	Leaf and tuber treatment	Fungal immunity	213
Gold nanoparticles (AuNPs)	CRISPR/Cas13a	Potato Virus Y (PVY)	Viral RNA (PVY)	RNA targeting system blocks viral replication	Foliar nanoparticle delivery	RNA virus interference	214

#### 5.2.2 Gene silencing

Nanobiotechnology has the ability to completely transform agricultural operations by improving the accuracy and effectiveness of pathogen management techniques and guaranteeing sustainable output in the face of changing phytopathogen threats. Potato phytopathogens, *Phytophthora infestans* and *Alternaria solani* severely impact crop yield, resulting in financial losses.^215^ The frequent use of synthetic chemicals against these phytopathogens has led to the development of resistance and environmental issues. Nanobiotechnology, a novel approach, has been applied to overcome pathogen resistance and environmental issues.^216^ Nanoparticles are also designed to deliver bioactive substances or RNAi molecules to target pathogens to replace their virulence genes precisely.^217^ For example, small interfering RNAs (siRNAs) targeting pathogen resistance genes can be encapsulated in gold or silver nanoparticles. Some studies have provided evidence about the focus of nanomaterials on silencing the genes of microbes related to their pathogenicity by the delivery of RNAi molecules. By acting as carriers, NMs protect RNAi molecules from unfavourable environmental conditions and enzymatic degradation, which affect gene silencing technology. The potato can be managed effectively. All these activities boost up the defense system of potato plants against microbes.^218^

Virus-specific nano-strategies can be tremendously useful in the treatment of potato viral diseases, especially PVY and PLRV. RNAi based on nanoparticles is carried out by utilizing siRNAs or dsRNAs that are viral in origin and are used to silence important viral genes, including those involving the coat protein and replication proteins. Polymeric, lipid, or inorganic nanoparticles are employed as nanoparticle carriers of RNA, resulting in greater antiviral efficacy and prolongation of antiviral duration. Nanoparticles may also be utilized to conjugate coat proteins of viruses, disrupting the assembly of the virus, its stability, or motility. Nano-based antivirals are more specific, less toxic to off-target and less prone to the development of resistance, making them the most suitable in the control of persistent and continually evolving potato viruses compared to traditional chemical-based antivirals.^219^

The targeted administration reduces detrimental effects on the environment through the efficient use of synthetic pesticides.^215,220^ Additionally, by employing nanocarriers, the stability and bioavailability of these medicines can be increased, guaranteeing prolonged release and better pathogen engagement. This is because the targeted mode of action can avoid conventional resistance routes. This strategy interferes with the life cycle of phytopathogens and reduces the chances of resistance emergence.^221^ Delivery methods mediated by nanoparticles are used to lower infection rates in potato crops. Furthermore, potato cultivars with improved resistance profiles can be developed by combining nanotechnology with conventional breeding and genetic engineering techniques.^147^ To achieve these advantages completely and handle any possible hazards related to the use of nanoparticles in agricultural systems, more in-depth research is vital.^215^

#### 5.2.3 Nanoscale interventions for resisting resistance: toward sustainable antimicrobials

**Fig. 5:**
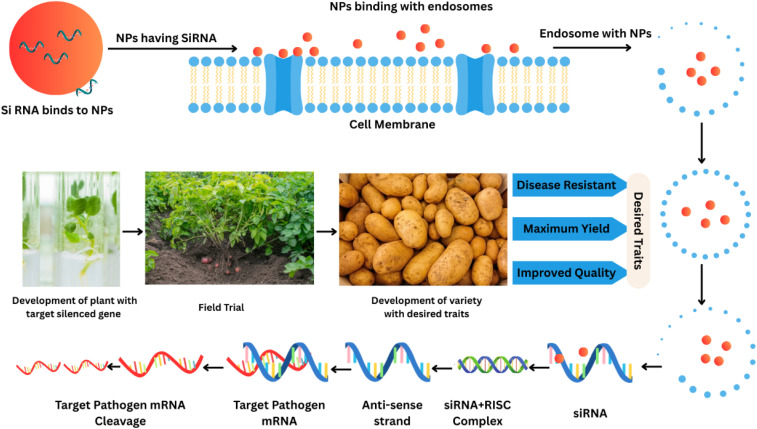
RNA interference (RNAi)-mediated gene silencing in plants *via* nanoparticle delivery systems. Engineered nanoparticles (NPs) functionalized with siRNA traverse the plant cell membrane *via* endocytosis, subsequently escaping endosomal compartments to avoid lysosomal degradation. Released siRNA strands integrate into the RNA-induced silencing complex (RISC), guiding the sequence-specific cleavage of complementary pathogen mRNA (anti-sense strand hybridization). Successful gene silencing confers pathogen resistance or desired phenotypic traits, validated through controlled field trials for stable varietal development.

Plant diseases include abnormalities in the structural and functional parts of plants due to continuous irritation by biotic or abiotic factors. Biotic factors, including microbes, like fungi, bacteria, viruses and nematodes, destroy the hopes of farmers of getting profitable crops in terms of quality and quantity.^222^ Due to the attack on these microbes, almost 30% of crop yields are lost, worth billions of dollars every year.^223^ By reducing these yield losses, the world may meet the needs of food for the increasing population. The potato crop is affected by abiotic and biotic factors; the latter cause losses up to 20% of the global potato production.^224^ This could be attained by imposing modern technologies in plant disease management. Among the modern technologies, nanotechnology is one of the most authentic, precise and sustainable technologies that is revolutionizing the agricultural sector.^225^ Nanotechnology focuses on two major domains of agriculture: crop production and crop protection. Crop production is increased by providing nanofertilizers and nanomaterial-based micro and macro-nutrients.^226^ However, a major increase in crop production is observed only when crops are well protected from the attack of pathogens. In the domain of crop protection, nanotechnology is working on diagnosing and curing pathogenic problems (Fig. 5).^132,227^

Although nanotechnology has a promising future in sustainable potato disease management, there are some limitations and environmental hazards that should be considered to ensure its better application in the field. The frequent application of engineered nanomaterials can result in their accumulation in agricultural soils, which can potentially affect soil physicochemical characteristics and positive microbial communities. Moreover, nanoparticles that are absorbed by the roots or deposited on foliage can be translocated into edible tubers, raising concerns about trophic transfer and long-term food safety. Some nanomaterials can also cause unexpected ecotoxicological impacts on non-target organisms in high concentrations due to oxidative stress or membrane damage. Hence, future research must focus on dose optimization, biodegradable or stimulus-responsive nanosystems, long-term field validation, and standardized regulatory motions so that the safety of the environment and acceptance by people are achieved.^228^

Another factor is whether long-term or repeated exposure to nanomaterials may lead to the development of resistance to plant pathogens. Compared with traditional agrochemicals, which often target sites, many nanomaterials have multi-modal antimicrobial effects such as physical disruption of the membranes, ion release and the production of reactive oxygen species, which decrease the chances of developing stable resistance. Nevertheless, these adaptive responses such as improvements in antioxidant defenses, biofilm formation or changed cell surface properties, cannot be eliminated entirely during long-term selective pressure. Consequently, nano-enabled strategies must be applied responsibly because dose optimization, rotation or combination with biological or conventional controls and avoiding chronic sub-lethal exposure may reduce the chances of resistance formation.^229^

## 6 Plant defense system: modulating innate immunity through nanomaterials

### 6.1 Activation of the defense system against microbiota

NPs enhance the immunological response of potato plants, resulting in the activation of the defense system. Roots or leaves of plants are immersed in NPs, like silica-, metal oxide (zinc oxide and copper)- and carbon-based nanomaterials.^230^ These NPs induce defense mechanisms through ROS production and serve as signaling molecules. ROS stimulate the plant's immune system depending on the defense-related genes that are activated.^226,231^ This improved immune response increases the production of defensive enzymes such as peroxidase, polyphenol oxidase and phytoalexins, which are naturally occurring antimicrobial compounds. Nanoparticles may also activate plant defense signaling pathways, including jasmonic acid and salicylic acid (SA), which induce PR genes to encode pathogenesis-related (PR) proteins that help protect plants against pathogen attack. This type of resistance is known as systemic acquired resistance (SAR).^232^ The SAR is the plant's natural defense system against bacteria, viruses and fungi, which is activated upon the attack of pathogens with no need for pesticide application.^233^ Besides ROS and hormone stimulation, nanoparticles trigger resistance *via* SA and JA signaling interactions rather than just their stimulation. Biotic resistance is typically associated with SA, and necrotrophic with JA, but the proportion of the two can be modulated by nanoparticle treatment based on the nanoparticle type, concentration, and mode of administration. Silica and metal oxide NPs can trigger SA signaling priming and tune genes sensitive to JA without antagonism and in concerted immunity.

Functional genetic analysis and gene expression confirm the immunity caused by nanoparticles. PR genes (PR1, PR2, and PR5) are consistently up-regulated in response to NP treatment; their functionality validates their contribution to the resistance caused by nanoparticles to differentiate between antimicrobial and immune responses in plants.^234^ The introduction of nanoparticles in agriculture has reduced the overuse of conventional pesticides, leading to a reduction in microbial resistance due to the repeated use of chemicals.^226^ In addition to activating the defense system, nanoparticles are used to deliver small amounts of fertilizer, insecticide or antimicrobial agents under a time-released system to ensure the death of the targeted pathogen with minimal input of chemicals. This reduces environmental pollution, lowers the chance of developing pest resistance and develops sustainable agriculture. Silica and carbon-based nanoparticles play a role in boosting the natural defense systems of plants. For example, silica nanoparticles (SiO_2_-NPs) may help potato plants by developing systemic acquired resistance (SAR) towards disease.^235^ For example, carbon nanotubes (CNTs) have been beneficially affecting plant growth and thence immunological response without heavy chemical treatment, aiding in the resistance of potatoes to microbial diseases.^236^

### 6.2 Plant defense systems against abiotic stresses

Nanosystems have the potential to improve the resistance of potato plants against three primary abiotic stresses: temperature (heat and cold), humidity (drought and waterlogging), and UV radiation. Despite abiotic stress tolerance seeming marginal to disease resistance, growing evidence suggests that abiotic stresses (drought, temperature extremes, and waterlogging) have a significant and detrimental effect on plant disease resistance through the disruption of physiological barriers and immune signaling pathways. Stress-induced disruption of redox homeostasis, membrane integrity and the hormonal signaling systems in potato has the potential to impair defense mechanisms, thus reducing the disease severity. Based on this, nano-mediated mitigation of abiotic stress can indirectly increase disease resistance by maintaining immune competence, cellular homeostasis and plant resilience under related stress conditions. Hence, the adaptation of abiotic stress control is directly connected to the development of disease-resistant potato systems with robustness.^237^ We describe the mechanisms behind the protection by nanoparticles and cite recent studies with an emphasis on sustainable potato production applications.

#### 6.2.1 Temperature stress

Potato is sensitive to temperature stress and is a cool-season crop. Temperatures above 25 °C can halt tuberization, and temperatures below −2 °C can lead to freezing injury. These stresses induce physiological disorders, like membrane damage and hormonal imbalance, lowering yield and quality. NPs enhance temperature stress tolerance through various mechanisms; they induce heat shock factors (HSFs) and proteins (HSPs) that shield proteins against denaturation.^238^ They enhance the activity of superoxide dismutase (SOD), catalase (CAT), and peroxidase (POD) to detoxify ROS. Nanoparticles ensure membrane integrity by reducing lipid peroxidation and influencing stress hormone balance, like ABA and ethylene, for stress responses.^239^

#### 6.2.2 Humidity stress

Humidity stresses in potato cultivation occur as droughts or waterlogging. Potatoes are water sensitive, where moderate drought leads to yield loss. Waterlogging leads to oxygen deficiency in the roots, impacting nutrient uptake and disease. Nanoparticles can modify root architecture, enabling adaptation to water stress. Aeroponic research indicates that certain nanoparticles produce longer, branching roots that tap more water. Field research indicates that drought-resistant cultivars develop longer roots with nanoparticles than with sensitive ones. High-throughput imaging has enabled the precise analysis of nanoparticle impacts on root morphology under water stress. Enhanced root structure from nanoparticle treatment enhances the plant's tolerance to drought and waterlogging by enabling greater water and oxygen access.^240^

#### 6.2.3 Nanosystems for UV protection

Ultraviolet radiation, particularly UV-B (280-315 nm), is increasingly becoming a source of concern in potato cultivation due to the depletion of the ozone layer. Low UV-B possibly indicates development, but higher exposure causes DNA harm and oxidative stress. Nonetheless, controlled UV can increase the desirable secondary metabolites in potatoes. Significantly, UV-stressed nanoparticles can increase the anthocyanin content in colored potatoes.^241^

Reciprocal grafting proved that potato tubers synthesize anthocyanins *in situ*, not from leaves. UV-B irradiation of higher intensity (2.5 kJ m^−2^ days^−1^) significantly enhanced total anthocyanin contents and some anthocyanidins (pelargonidin and peonidin) in red-fleshed potatoes. Transcriptome analysis indicated upregulation of anthocyanin-related enzymatic genes (PAL, C4H, 4CL, CHS, CHI, F3H, F3′5′H, ANS, UFGTs, and GSTs) with UV-B treatment. Transcription factors, like StbHLH1 and several MYB factors, were induced at increased expression under UV-B, controlling anthocyanin biosynthesis. Low-dose UV-B treatment (5 kJ m^−2^) promoted the expression of anthocyanin structural genes without altering agronomic traits, while higher doses (10 kJ m^−2^) damaged plants and reduced yield. This emphasizes the need to optimize nanoparticle and UV exposure for desirable effects without stress damage.

### 6.3 Nanosystems trigger general defense mechanisms

Nanoparticles induce potato plant defense in various ways. They enhance enzymatic antioxidant defense (SOD, CAT, APX, and GPX) and boost non-enzymatic antioxidants (ascorbate, glutathione, phenols, and flavonoids). This enhances the suppression of ROS from abiotic stress. Nanoparticles trigger osmolyte production such as proline, glycine betaine, and sugars, which maintain turgor and protect macromolecules.^236^ They affect stress hormone signaling (ABA, ethylene, and jasmonic acid) and trigger calcium signaling for stress. Nanoparticle treatment can precondition defenses for quicker responses to future stresses. They maintain chlorophyll retention and photosystem II activity, protecting thylakoid membranes and retaining photosynthesis for recovery.^242^

### 6.4 Omics

Recent technological advancements in omics technologies have improved the understanding of nanoparticle-induced stress tolerance. Nanoparticle treatment controls gene expression related to perception, signaling, and response to stress. Important pathways controlled are MAPK signaling, phenylpropanoid biosynthesis, and hormone metabolism. NMs control stress-related proteins (HSPs, dehydrins, and ROS-scavenging enzymes) and protein phosphorylation, influencing functionality. NMs also reorganize metabolite profiles, sustaining stress tolerance and protective compounds such as anthocyanins, flavonoids, and phenolic acids. Nanoparticles can induce DNA methylation changes in gene expression, while histone modifications can determine stress-responsive chromatin states. These omics strategies reveal the intricate control of nanoparticles on plant stress responses, moving away from phenomenology to mechanistic understanding.^241^

## 7 Conclusion

The extensive use of pesticides to control diseases plays a role in the development of pathogen resistance. Nanobiotechnology has played a significant role in conferring microbial resistance and enhancing disease resistance in potato plants through the manipulation of different nanomaterials at the nanoscale. As carriers, NMs organize the controlled release of drug delivery against the attack of pathogens in order to mitigate the effects of synthetic chemicals, which deteriorate the environment. NMs have proven themselves to be shields against pathogens and their chemical-based management strategies in order to conserve biodiversity. In addition to disease protection in the potato crop, they have offered extensive roles to be used as carriers of RNAi in order to silence the virulence genes of microbes. Despite playing an adventitious role in modern plant disease management, the field still needs a lot of improvement. The delivery and penetration of NMs in both plant and microbial cells need to be approved because both depend on the size and the charge of NMs. There is a lot of research gap existing on defining the cost of synthesis and applications, their uptake by plants, their stability and solubility, shelf life and other properties of NMs that should be the focus of researchers and nano-scientists. Environmental safety, regulatory frameworks and phytotoxicity concerns still need discussions to improve them. All these improvements will be very important in describing the ability of nanobiotechnology to revolutionize agriculture, assure global food safety and develop sustainability.

## 8 Future directions and challenges

The promising future of nanobiotechnology in plant disease management with several directions for research and development includes the development of multifunctional NMs like targeted delivery as a carrier for disease detection, disease treatment, nutrient supplementation, and as an immunity booster simultaneously in plants to improve the plant health status. The development of nanobiosensors for disease detection and the reduction of chemical usage and dosage for disease treatments provide enough areas for future directions. Environmental protection, dose-dependent phytotoxicity, and regulatory provisions need further research before the application of nanoparticle-based formulations in agriculture on a large scale. Future studies need to focus on long-term plant safety research, setting of crop-specific exposure limits and alignment with new regulatory standards to enable nano-enabled plant protection technologies to be both effective and environmentally friendly. All these future directions lead nanobiotechnology to greater precision, stability and effectiveness for plant disease management strategies. The difficulty in the handling and preparation of NMs on a large scale, their side effects when applied on plants and the challenges in the evaluation of risk assessments for their use are the major obstacles that hinder the adoption of nanobiotechnology as a plant disease management strategy. Future studies must focus on the mechanistic degradation of immune responses induced by nanoparticles using functional genomic methods. Studies with hormone signaling mutants, PR gene knockouts, and CRISPR/Cas editing of key defense regulators will be crucial to understand SA–JA interactions and establish causal defense pathways. The integration of transcriptomics, proteomics, and metabolomics analyses with gene perturbation provides a system-level understanding of disease resistance triggered by nanoparticles, allowing for the sensible design of new-generation nano-enabled crop protection methods. To further enhance translational potential, future studies must focus on designing biodegradable and stimulus-responsive nanomaterials that can degrade or activate in response to particular biological or environmental signals, thus minimizing environmental persistence. Moreover, the integration of nanosensor platforms with AI and machine learning (ML) models may enable real-time disease surveillance, predictive disease forecasting, and precise nano-enabled intervention deployment. These data-driven and adaptive plant protection systems represent an essential next step towards sustainable and intelligent disease-resistant potato production.

## Conflicts of interest

The authors declare no conflicts of interest.

## Abbreviations

NMs: Nanomaterials
NPs: Nanoparticles
CNTs: Carbon nanotubes
QD: Quantum dot
CS-NPs: Chitosan nanoparticles
LNPs: Lipid-based nanoparticles
AuNPs: Gold nanoparticles
SiO_2-_NPs: Silica nanoparticles
MSNs: Mesoporous silica nanoparticles
LDH: Layered double hydroxide
ROS: Reactive oxygen species
PR: Pathogenesis-related
RNAi: RNA interference
siRNAs: small interfering RNAs
CRISPR: Clustered regularly interspaced short palindromic repeats
Cas9: CRISPR-associated protein 9
ZFNs: Zinc finger nucleases
TALENs: Transcription activator-like effector nucleases
SHREDDER: Short hairpin RNA enhancing degradation of disease-associated exons *via* RNA interference
FAO: Food and agriculture organization
KPK: Khyber Pakhtunkhwa
TDAP: Trade development authority of Pakistan
TEM: Transmission electron microscopy
SEM: Scanning electron microscopy
SOD: Superoxide dismutase
CAT: Catalase
POD: Peroxidase
APX: Ascorbate peroxidase
GPX: Glutathione peroxidase
MAPK signalling: Mitogen-activated protein kinase
HSFs: Heat shock factors
HSPs: Heat shock proteins
MAMPs: Microbe-associated molecular patterns
PAMPs: Pathogen-associated molecular patterns
PRRs: Pattern recognition receptors
PTI: Pattern triggered immunity
ETI: Effector triggered immunity
HR: Hypersensitive response
SAR: Systemic acquired resistance
PEI: Polyethyleneimine
NHEJ: Non-homologous end joining
HDR: Homology-directed repair
DMR: Differentially methylated region
NLRs: Nucleotide-binding domain and leucine-rich repeat-containing receptors

## Data Availability

No primary research results, software or code have been included and no new data were generated or analyzed as part of this review.
